# Supplementary material for: A Comparison of the Impact of Restrictive Diets on the Gastrointestinal Tract of Mice
Source: Nutrients. 2022 Jul 29;14(15):3120. doi: 10.3390/nu14153120 (PMC9370610; doi:10.3390/nu14153120)
Supplement: Supplementary file 1 [file nutrients-14-03120-s001.zip › nutrients-1827538-SM.pdf]

Supplementary figures

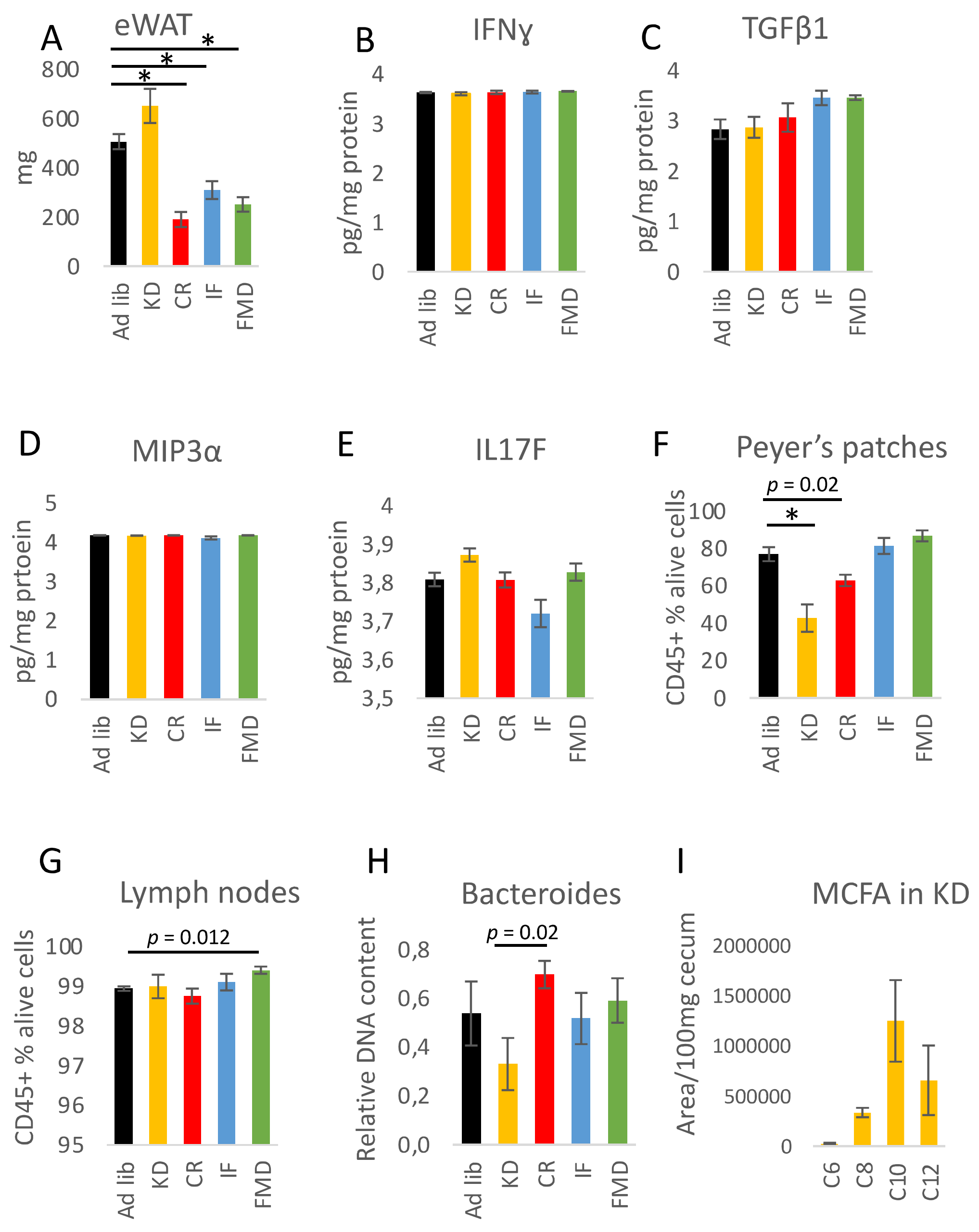

Supplementary figure S1. WAT weight, protein expression, cells abundance, Bacteroides abundance, and MCFA levels. The weight of epididymal white adipose tissue (WAT) was verified during dissection (A). The concentration of IFNɣ (B), TGFβ1 (C), MIP3α (D), and IL17F (E) was measured by applying protein arrays. The percentage of CD45%+ cells in Peyer's patches (F) and lymph nodes (G) was measured with FACS. The relative abundance of Bacteroides was assessed using qRT-PCR (H). Medium-chain fatty acids (MCFA) levels were measured in mice cecum content by applying cecum HPLC-MS/MS. The groups were compared using ANOVA followed up by Student's t-test with correction for multiple testing. Considering the correction $p<0.01$ was regarded statistically significant; n=6-8. Error bars indicate ±SEM.

A

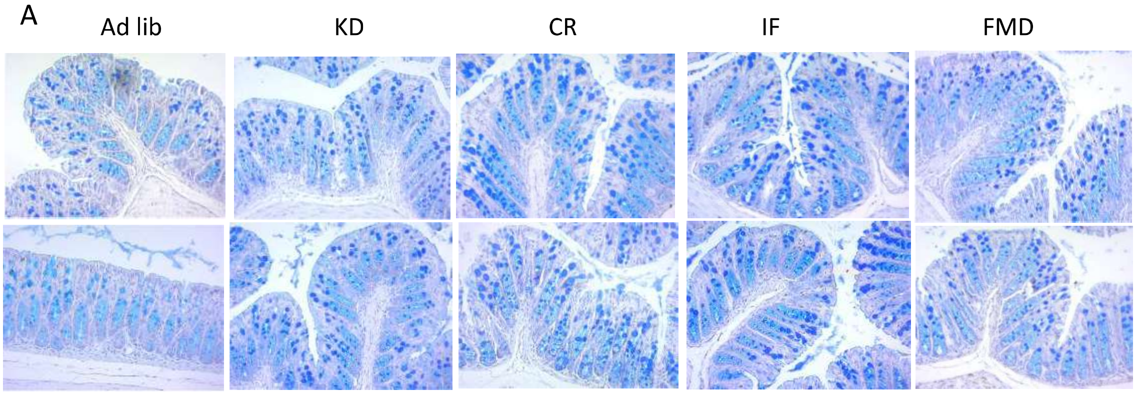

B

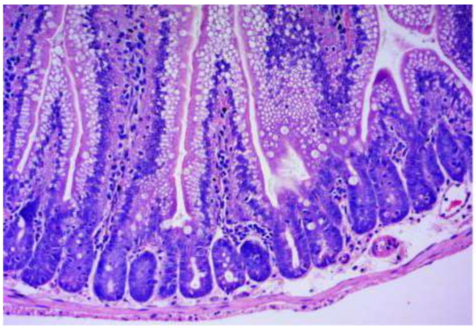

Supplementary figure S2. Additional histology pictures. Histology sections of the colon were stained with alcian blue periodic acid-Shiff (AB-PAS) to visualize goblet cells and mucus (A). Histological sections of jejunum were stained with hematoxylin-eosin (B).

Supplementary tables

Name	*Forward*	*Reverse*
Akkermansia	GCGTAGGCTGTTTCGTAAGTCGTGTGTGAAAG	GAGTGTTCCCGATATCTACGCATTTCA
Atg7	GTTCGCCCCCTTTAATAGTGC	TGAACTCCAACGTCAAGCGG
Atg12	TAGAGCGAACACGAACCATCC	CACTGCCAAAACACTCATAGAGA
Catalase	CACTGCCAAAACACTCATAGAGA	GTAGAATGTCCGCACCTGAG
Deferribacteres	CTATTTCCAGTTGCTAACGG	GAGATGCTTCCCTCTGATTATG
Firmicutes	TGAAACTYAAAGGAATTGACG	ACCATGCACCACCTGTC
Gsta3	GTAGAATGTCCGCACCTGAG	GCATGGCGGTACAAGCCTTT
Ifnɣ	ATGAACGCTACACACTGCATC	CCATCCTTTTGCCAGTTCCTC
Irf1	CCCAGCTCTTGCTTTCGGA	AAGCCCAGTAGTTCACGACC
Lactobacillus	AGCAGTAGGGAATCTTCCA	CACCGATACACATGGAG
Lc3	GTCCTGGACAAGACCAAGTTCC	CCATTCACCAGGAGGAAGAAGG
Il1α	CAAGATGGCCAAAGTTCGTGAC	GTCTCATGAAGTGAGCCATAGC
Il1β	TCCTGTGTAATGAAAGACGGC	GGTGCTGATGTACCAGTTGGG
Il6	TAGTCCTTCCTACCCCAATTTCC	TTGGTCCTTAGCCACTCCTTC
Il7	TCTGCTGCCTGTCACATCATC	GGACATTGAATTCTTCACTGATATTCA
Il33	TGAGACTCCGTTCTGGCCTC	CTCTTCATGCTTGGTACCCGAT
Mgst1	CCTTCTCCCTGGATTCAGTCAT	TCGGCCATGCTTCCAATCTT
MnSOD	TGGCTTGGCTTCAATAAGGA	AAGGTAGTAAGCGTGCTCCCACAC
Mt2	CCGCTATAAAGGTCGCGCT	AGGAGCAGGATCCATCGGAG
mtDNA	CATCTGGTTCCTACTTCAGGG	TGAGTGGTTAATAGGGTGATAGA
Muc2	CAAGGGCTCGGAACTCCAG	CCAGGGAATCGGTAGACATCG
Muc13	GCTACAGTGGAGTTGGCTGT	GACGAATGCAATCACCAGGC
MyD88	GCACCTGTGTCTGGTCCATT	TGTTGGACACCTGGAGACAG
Nod2	GGCAACAGTGTAGGTGATAAGGG	TAGTGACTTGTTCTTCTCCAGCATC
Oas1a	ATGGAGCACGGACTCAGGA	TCACACACGACATTGACGGC
Occludin	CCTCCAATGGCAAAGTGAAT	CTCCCCACCTGTCGTGTAGT
Parasutterella	AACGTRTCCGCTCGTGGGGGAC	CGGAATAGCTGGATCAGGCTTG
Pgc1α	GCGTCATTCGGGAGACTGGAT	CCAACCAGAGCAGCACACTCT
Reg3ɣ	CTCCCCACCTGTCGTGTAGT	CTCCCCACCTGTCGTGTAGT
Rsad2	TGCTGGCTGAGAATAGCATTAGG	GCTGAGTGCTGTTCCCATCT
Stat1	GCTGAGTGCTGTTCCCATCT	AAGTCCTTCAGAGTAACAG
Tfam	TCCACAGAACAGCTACCCAA	CCACAGGGCTGCAATTTTCC
Tgfβ1	CCACAGGGCTGCAATTTTCC	CCACAGGGCTGCAATTTTCC
Tlr3	GTATTGCCTGGTTTGTTAATTGG	AAGAGTTCAAAGGGGGCACT
Tnfα	CCACAGGGCTGCAATTTTCC	CCACAGGGCTGCAATTTTCC
Trx2	GCTAGAGAAGATGGTCGCCAAGCAGCA	TCCTCGTCCTTGATCCCCACAAACTTG
Ucp2	CAGTTCTACACCAAGGGCTCAGAG	TGACAATGGCATTACGGGCAACAT
Zo-1	CCACCTCTGTCCAGCTCTTC	CACCGGAGTGATGGTTTTCT

Supplementary table S1. Primer sequence used during the study.
